# Mixed Mode Fracture Investigation of Rock Specimens Containing Sharp V-Notches

**DOI:** 10.3390/ma15248779

**Published:** 2022-12-08

**Authors:** Ali Arabnia, Javad Akbardoost, Sergio Cicero, Ali Reza Torabi

**Affiliations:** 1 Department of Mechanical Engineering, Faculty of Engineering, Kharazmi University, Mofatteh Avenue, Tehran P.O. Box 15719-14911, Iran; 2 LADICIM (Laboratory of Materials Science and Engineering), E.T.S. de Ingenieros de Caminos, Canales y Puertos, Universidad de Cantabria, Av/Los Castros 44, 39005 Santander, Spain; 3 Fracture Research Laboratory, Faculty of New Sciences and Technologies, University of Tehran, Tehran P.O. Box 14395-1561, Iran

**Keywords:** rock fracture, sharp V-shaped notch, mixed mode I/II, finite element method, MTS-FEM criterion

## Abstract

This work aims to assess both experimentally and analytically the fracture behavior of rock specimens containing sharp V-notches (SV-notches) subjected to mixed mode I/II loading. To this end, firstly, several mixed mode fracture tests were conducted on Brazilian disk specimens weakened by an SV-notch (SVNBD sample), performed in their corresponding center and with various notch opening angles. Secondly, the fracture resistance of the tested samples was predicted using a criterion named MTS-FEM. This approach is based on the maximum tangential stress (MTS) criterion, in which the tangential stress is determined from the finite element method (FEM). Additionally, in the present research, the required critical distance is calculated directly from finite element analyses performed on cracked samples. Comparing the experimental results and the analytical predictions, it is shown that the fracture curves obtained from the MTS-FEM criterion are in agreement with the experimental results. These results are achieved without the need for the calculation of stress series expansion coefficients, as an additional advantage of the proposed approach.

## 1. Introduction

The existence of different natural defects such as holes, grain boundaries, pores, cracks, and notches define the mechanical behavior of rock masses. Moreover, diverse artificial notch-like defects with different shapes may be created in rock masses when dams or tunnels are built, or when gas and oil wells and excavations are performed. All these defects act as stress risers and play a key role in fracture initiation processes. Therefore, the fracture of notched and cracked samples made of rock has been extensively investigated in the literature. For this purpose, fracture mechanics, which assess the fracture resistance of cracked or notched parts, have been frequently utilized. Since the stress concentration in the sharp V-notch (SV-notch) is more pronounced than that caused by other types of notches, this shape is more dangerous for rock structures, making the corresponding prediction of the load-bearing capacity in rock samples containing SV-notches even more imperative.

In the context of fracture mechanics, there are two main failure modes for notched specimens: (1) pure mode I, or opening mode, in which the notch flanks open without any sliding; and (2) pure mode II, or in-plane shearing mode, in which the notch flanks slide relative to each other without any opening or closing. Generally, due to the arbitrariness of the applied load direction relative to the notch orientation, the combinations of opening and in-plane shearing modes can appear in notch samples. Although several brittle fracture criteria can be found in the fracture mechanics literature for predicting the fracture load of SV-notched samples, three criteria have received more attention: the strain energy density (SED) [1,2,3,4], the maximum tangential stress (MTS), and the mean stress (MS) [5,6,7,8] criteria. A brief explanation of the studies dealing with these three criteria for investigating the fracture resistance of SV-notched samples is described here: the crack initiation angle in V-notched samples under biaxial loading has been investigated by Seweryn and Lukaszewicz [9], using the SED criterion in addition to the other fracture criteria. Kim and Cho [10] suggested a unified brittle fracture criterion for predicting the mixed mode fracture resistance of cracked and V-notched samples by extending the MTS and Novozhilov’s criteria. An approach developed according to the MTS criterion has been proposed by Ayatollahi et al. [8] to anticipate the brittle fracture in engineering components containing SV-notches under mixed mode I/II loading. In additional studies, Ayatollahi et al. [11] assessed the brittle fracture of polycrystalline graphite by testing Brazilian disks containing sharp and rounded-tip V notches (RV notches) subjected to different loading angles. Then, they utilized an average value of the strain energy density over a well-defined volume to predict the resistance of the tested specimens. In this way, there are other research works in the literature to calculate numerically and experimentally the notch stress intensity factors (NSIFs) describing the fracture resistance of notched samples. Ayatollahi and Nejati [12] employed the photoelasticity technique for determining the values of NSIFs in SV-notched components. Using the digital image correlation method, Bahrami et al. [13] obtained the values of NSIFs for diagonally loaded square plates having SV-notch under pure mode I, mixed mode I/II and pure mode II loading. The NSIF parameters have been determined for SV-notched sample by Paul et al. [14] utilizing robust and simple single strain gages. In addition to experimental methods, there are a number of studies that deal with the calculation of NSIFs. Ayatollahi and Nejati [15] proposed a technique named finite element over-deterministic method for obtaining NSIFs for SV-notched parts, as well as the coefficients of higher order terms in the stress series expansion. The fractal finite element method has been employed by Treifi et al. [16] to determine the NSIFs for SV-notched samples. It should be noted that, in almost all these studies, the stress, the strain or the displacement fields are first determined experimentally and numerically for some specific points around the notch border. Then, a set of linear equations (in which the NSIFs are unknown) are derived according to formulations proposed by the aforementioned fields (for example, the relations derived by Williams [17] for stress components around the SV-notch tip). The NSIFs are finally obtained by solving these linear equations simultaneously.

From the point of view of fracture assessment in notched rock specimens, there are a few studies in the literature that are, in any case, much less common than those found for other brittle materials. For instance, the mode I fracture resistance of U-notched beams made of various rock types has been investigated by Justo et al. [18,19,20] experimentally and theoretically. Ghadirian et al. [21] carried out a set of experiments on Brazilian disks with central RV and U-shaped notches and then predicted the onset of fracture for the tested samples using a modified version of the MTS criterion. Sangsefidi et al. [22] attempted to estimate the mixed-mode fracture resistance of rock-type U-notched specimens by means of the MTS criterion in conjunction with the finite element method (FEM). Since there is no study in the literature that deals with the fracture behavior of rock SV-notched parts, the aim of this study is to assess experimentally and theoretically the fracture resistance of marble SV-notched samples under mixed mode I/II loading. First, a number of Brazilian disks containing a centered SV-notch (SVNBD samples) were manufactured from white marble and then tested under mixed mode loading. After that, the fracture loads obtained from the fracture tests were predicted using the MTS criterion, in conjunction with FEM. The theoretical approach utilized in this study is similar to the MTS criterion proposed previously by Sangsefidi et al. [22] for U-notched samples, but is extended here for SV-notched specimens. The proposed approach, namely the MTS-FEM criterion, is a particular form of the MTS criterion in which the tangential stress around the SV-notch border is determined using finite element analysis. In addition, the critical distance, which is an important parameter in MTS-FEM criterion, is calculated directly from FEM. It is revealed that the results predicted by the MTS-FEM criterion are in agreement with those obtained from testing SVNBD specimens.

## 2. Materials and Methods

### 2.1. Material, Tests and Experimental Results

As mentioned above, there were no experimental data for investigating the mixed mode fracture resistance of rock SV-notched samples. Hence, in this work several Brazilian disks containing a central SV-notch (SVNBD samples) were prepared and tested under mixed mode I/II loading.

**Figure 1:**
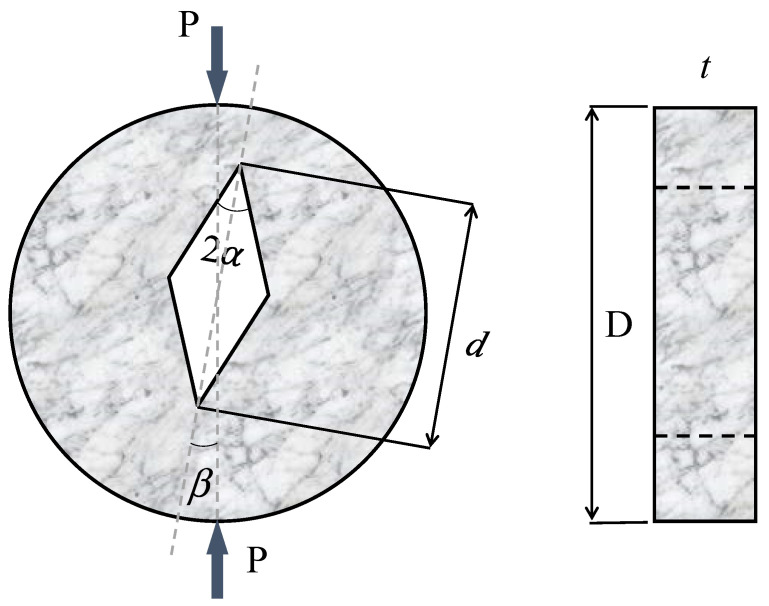
Schematic of SVNBD samples under mixed mode I/II loading.

As shown schematically in Figure 1, the SVNBD specimen is a circular disk with dimeter and thickness *D* and *t*, respectively. There is a central rhombus with the large diagonal being *d* and the corner angle being 2*α*. The upper and lower corners of the rhombic hole in the SVNBD samples can be considered sharp V-notches. A compressive load (*P*) is applied along the diameter of the disk whose direction relative to the notch bisector line, i.e., the loading angle *β*, defines the mode mixity ratio. The alignment of the applied load with the notch bisector line, i.e., *β =* 0, gives the pure mode I loading. By rotating the notch bisector line relative to the applied load, the loading condition changes from pure mode I to mixed mode I/II, until a specific angle *β_II_*, for pure mode II loading, is achieved. Torabi and Taherkhani [23] calculated the values of *β_II_* as a function of the notch length ratio (d/D) and the notch opening angle 2*α* using FEM. The dimensions and loading conditions of the SVNBD samples tested in the present study are listed in Table 1. It is noteworthy that the loading angle *β* for pure mode II (i.e., *β_II_*) for each notch opening angle was extracted from the study carried out by Torabi and Taherkhani [23]. Additionally, the pure mode II loading angle *β_II_* depends on the notch angle 2*α* and, therefore, the values of *β_II_* for notch opening angles 2*α* = 30°, 60°, and 90° are different. The loading angles *β* in mixed mode loading were defined accordingly to the value of *β_II_* for each opening angle 2*α*.

**Figure 2:**
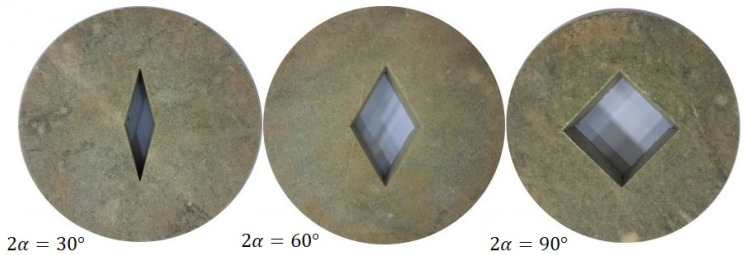
SVNBD samples with notch angles (2*α*) of 30°, 60° and 90° manufactured for mixed-mode fracture tests.

**Figure 3:**
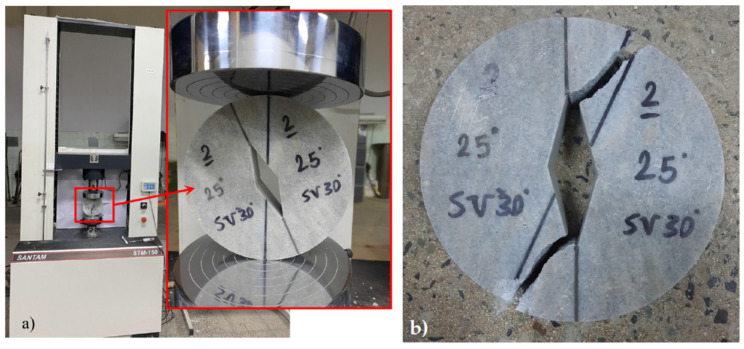
SVNBD sample with 2*α* = 30° under pure mode II loading; (**a**) inside the test machine; (**b**) after fracture.

The specimens were manufactured from white marble sheets with a mean thickness of 30 mm and by means of a water jet machine. The central rhombic hole was also created by water jet machining, with the notch corners sharpened by a razor blade. Figure 2 shows the SVNBD samples with different notch angles prepared for the fracture tests. In order to obtain reliable data, four repetitions were considered for each test category. Accordingly, 48 SVNBD samples were finally tested. After manufacturing the SVNBD samples, corresponding to the dimensions given in Table 1, they were tested in a SANTAM universal test machine (SANTAM company, Tehran, Iran) with a load cell capacity of 150 kN, under displacement control conditions, and with a cross head speed of 0.5 mm/min. Figure 3a shows, as an example, a SVNBD specimen with notch angle 30° subjected to pure mode II loading inside the test machine, while Figure 3b shows the corresponding broken halves. The fracture loads for each test sample were recorded from the test machine. The average fracture loads for each test category, as well as the standard deviations are listed in Table 1.

**Figure 4:**
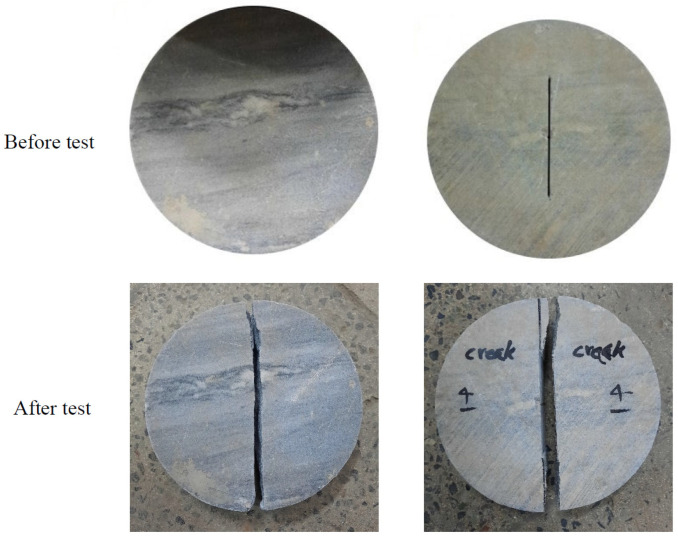
BD (**left**) and CBD (**right**) samples tested to determine the tensile strength and the fracture toughness, before and after being tested.

In addition to the SVNBD samples, Brazilian disk (BD) samples without any type of crack/notch, and cracked Brazilian disk (CBD) samples were tested in order to determine, respectively, the tensile strength and fracture toughness of white marble. Both mechanical properties were utilized in the theoretical estimations of the fracture loads. The diameter and thickness of both types of samples were, respectively, 150 mm and 30 mm. Furthermore, the crack length for all CBD samples was 2*a* = 75 mm. Figure 4 displays examples of BD and CBD samples before and after the tests. The average of the fracture loads for the BD samples, obtained from four test repetitions, was 28,061 N, while the average value for CBD samples (also four repetitions) was approximately 17,317 N. These two averages were the only mechanical inputs required to calculate the tensile strength and the fracture toughness, respectively.

The main results determined from the experiments are tensile strength (BD samples), fracture toughness (CBD samples), and critical notch stress intensity factors (SVNBD samples). The tensile strength *f_t_* can be written in terms of fracture load *P_f_*, diameter *D*, and thickness *t* of BD sample as [24]:

(1) $$f_{t}=\frac{2\cdot P_{f}}{\pi \cdot D\cdot t}$$

By substituting the values of *D* = 150 mm, *t* = 30 mm, and *P_f_* = 28,061 N in Equation (1), the value of the tensile strength for the tested marble is determined as *f_t_* = 3.34 MPa.

To calculate the fracture toughness of the analyzed marble, Equation (2) was utilized in this study [25]:

(2) $$K_{Ic}=\frac{2P_{f}}{Dt}\sqrt{\pi D}K_{I}^{*}$$

where the dimensionless parameter $K_{I}^{*}\;$ is usually obtained from finite element analyses and depends on the crack length ratio 2*a/D*. The value of this parameter for CBD samples with 2*a/D* = 0.5 is $K_{I}^{*}$ = 0.221, was extracted from the study performed by Akbardoost and Ayatollahi [25]. Substituting the dimensions of the CBD samples, the fracture load, and the dimensionless parameter $K_{I}^{*}$ into Equation (2), the fracture toughness is *K_Ic_* = 0.977 MPa√m.

Finally, $K_{\mathrm{If}}^{SV}\;$ and $K_{\mathrm{IIf}}^{SV}$ are the critical notch stress intensity factors (NSIFs) for mode I and mode II loading conditions, extensively utilized in the literature to describe the fracture resistance of SV-notched specimens against brittle fracture. The values of NSIFs for SVNBD samples, as well as for other V-notched (rounded or sharp tip) samples, can be obtained from the stress, strain or displacements fields determined for some specific points around the notch border, which can be derived numerically (e.g., finite element method [15,16]) or experimentally (e.g., digital image correlation [13], use of strain gauge [14], photoelasticity [12], etc.). After that, a set of linear equations in which the NSIFs are unknown are obtained according to the formulations proposed for the aforementioned fields (for example, the relations derived by Williams [17] for stress components around the SV-notch tip). Solving these linear equations simultaneously allows the values of NSIFs to be obtained. Torabi and Taherkhani [23] employed the finite element method for determining the stress field around the rounded and sharp V-notched Brazilian disks (RVNBD and SVNBD samples), and calculated the corresponding mode I and mode II NSIFs for a wide range of geometrical ratios and loading conditions. For the sake of more usability, they proposed Equations (3) and (4) for SVNBD samples:

(3) $$K_{\mathrm{If}}^{SV}=\frac{P_{f}}{Dt}{\left(d\right)}^{1-\lambda_{1}}Y_{I}^{SV}$$

(4) $$K_{\mathrm{IIf}}^{SV}=\frac{P_{f}}{Dt}{\left(d\right)}^{1-\lambda_{2}}Y_{\mathrm{II}}^{SV}\;$$

**Figure 5:**
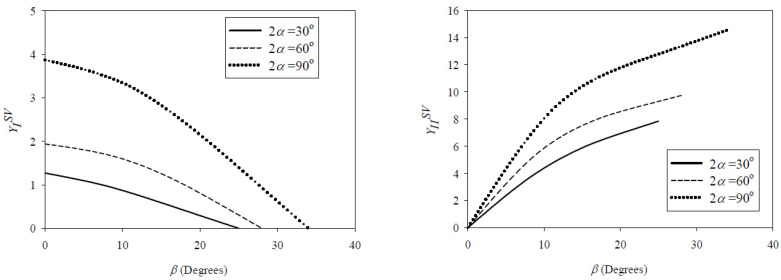
Variations of *Y_I_^SV^* and *Y_II_^SV^* vs. loading angle for SVNBD samples [23].

$Y_{I}^{SV}$ and $Y_{\mathrm{II}}^{SV}$ are dimensionless parameters depending on the relative notch length *d/D*, the loading angle *β*, and the notch angle 2*α*. Their values for the SVNBD samples tested in the present work were extracted from the study performed by Torabi and Taherkhani [23], and are shown in Figure 5, and listed in Table 2. *λ*_1_ and *λ*_2_ are eigenvalues of the stress field near the SV-notch border, and only depend on the notch angle 2*α*. Their values for notch angles of 30°, 60°, and 90° were obtained from [26], as shown also in Table 2. Now, the values of $K_{\mathrm{If}}^{SV}$ and $K_{\mathrm{IIf}}^{SV}$ for the tested SVNBD samples were calculated by replacing the corresponding values of the average fracture load, specimen dimensions, eigenvalues *λ*_1_ and *λ*_2,_ and dimensionless parameters $Y_{I}^{SV}$ and $Y_{\mathrm{II}}^{SV}$, as gathered in Table 2. It should be noted that the units of $K_{\mathrm{If}}^{SV}$ and $K_{\mathrm{IIf}}^{SV}$ are calculated in terms of eigenvalues *λ*_1_ and *λ*_2_, which depend on the notch opening angle 2*α* (as easily observed from Equations (3) and (4)). Therefore, unlike the crack stress intensity factor, whose dimension is the same for both mode I and mode II SIFs, the units of NSIFs for mode I and mode II loading conditions in SV-notched samples are meaningfully different.

In Section 3, the values of $K_{\mathrm{If}}^{SV}$ and $K_{\mathrm{IIf}}^{SV}$ will be predicted by using the MTS-FEM criterion.

### 2.2. Analytical Approach: Mixed-Mode Fracture Criterion

**Figure 6:**
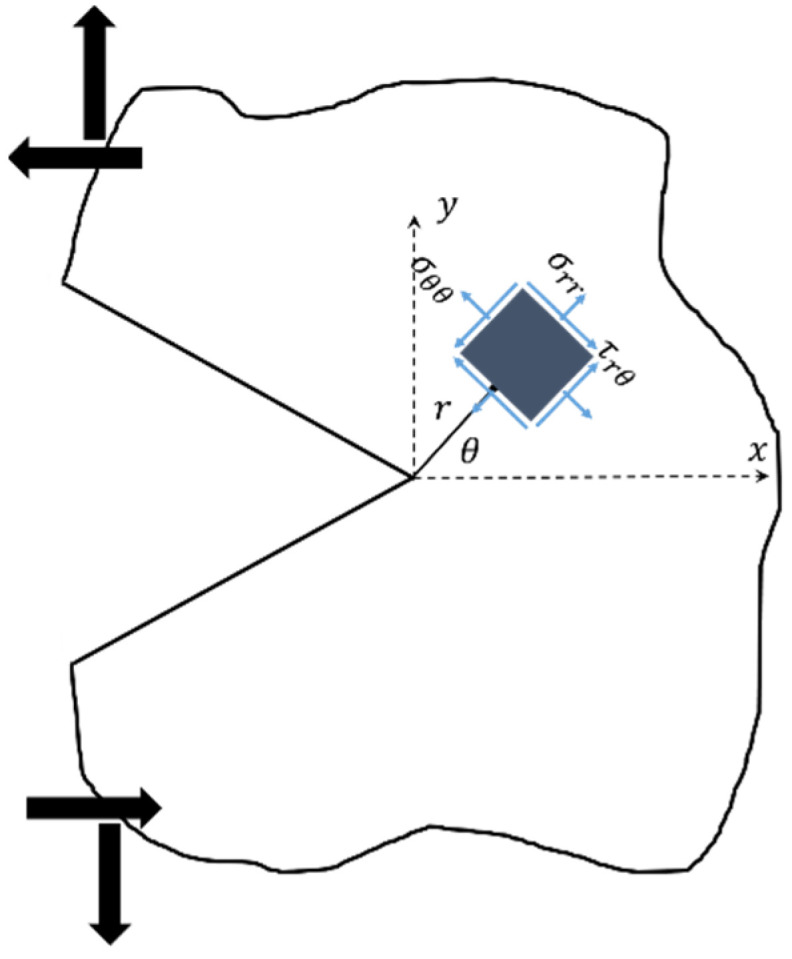
A sharp V-notch with its corresponding coordinates.

Like other quasi-brittle materials, rock materials have low resistance under tensile loading and, hence, rock components often fail perpendicularly to the maximum tensile principal stress. Thus, fracture criteria based on this observation provide good estimates for the onset of fracture in rock structures. The maximum tangential stress (MTS) criterion is among these fracture criteria. In the MTS criterion, brittle fracture in cracked or notched specimens takes place when the value of the tangential stress *σ_θθ_*, at a specified critical distance from the crack or the notch tip, attains the critical value *σ_θθc_*. Also, the crack growth progresses from the defect tip perpendicularly in the direction of the maximum tangential stress. In order to apply the MTS criterion, it is necessary to determine the tangential stress component around the notch tip. Therefore, the relations for determining the stress components around the SV-notch tip are taken into consideration. A sharp V-shaped notch with a coordinate system originating at the notch tip is shown in Figure 6. Williams [17] made use of the Airy stress function method and derived the elastic stresses around a sharp V-notch. By simplifying the parameters and functions described by Ayatollahi et al. [8], the tangential stress component around the SV-notch border can be rewritten as:

(5) $$\begin{array}{cc} \sigma_{\theta \theta }= & K_{I}^{SV}r^{-n}\left[\left\{-\cos\left(m\theta \right)+\left(\frac{m}{n}\right)\frac{\sin\left(\omega m/2\right)}{\sin\left(\omega n/2\right)}\cos\left(n\theta \right)\right\}/\sigma_{\theta \theta }^{I}\left(\theta =0\right)\right]\\ & +K_{\mathrm{II}}^{SV}r^{-q}\left[\frac{\left\{-\sin\left(p\theta \right)+\frac{\sin\left(\frac{\omega p}{2}\right)}{\sin\left(\frac{\omega q}{2}\right)}\sin\left(q\theta \right)\right\}}{\sigma_{r\theta }^{II}\left(\theta =0\right)}\right]+higher\;order\;terms \end{array}$$

where $K_{I}^{SV}$ and $K_{II}^{SV}$ are the notch stress intensity factors for mode I and mode II, and *r* and *θ* are the polar coordinates, respectively. Additionally:

(6) $$\begin{array}{c} \omega =2\pi -\alpha \\ m=1+\lambda_{1}\\ n=1-\lambda_{1}\\ p=1+\lambda_{2}\\ q=1-\lambda_{2} \end{array}$$

(7) $$\sigma_{\theta \theta }^{I}\left(\theta =0\right)=\frac{m}{n}\frac{\sin\left(\omega m/2\right)}{\sin\left(\omega n/2\right)}-1$$

(8) $$\sigma_{r\theta }^{II}\left(\theta =0\right)=1-\frac{q}{p}\frac{\sin\left(\omega p/2\right)}{\sin\left(\omega q/2\right)}$$

Table 2 above summarizes the values of the parameters *λ*_1_ and *λ*_2_ for the SVNBD specimens with different notch opening angles, as provided by Ayatollahi et al. [8]. The same authors [8] employed only the first or singular terms of Equation (5), and proposed the SV-MTS criterion for predicting $K_{If}^{SV}$ and $K_{IIf}^{SV}$ in SV-notched samples as follows:

(9) $$\left(K_{If}^{SV}/K_{\mathrm{Ic}}^{SV}\right)\left[\left\{-\cos\left(m\theta_{0}\right)+\left(\frac{m}{n}\right)\frac{\sin\left(\omega m/2\right)}{\sin\left(\omega n/2\right)}\cos\left(n\theta_{0}\right)\right\}/\sigma_{\theta \theta }^{I}\left(\theta =0\right)\right]+\left(K_{\mathrm{IIf}}^{SV}/K_{\mathrm{Ic}}^{SV}\right){\left(r_{c,V}\right)}^{t}\left[\left\{-\sin\left(p\theta_{0}\right)+\frac{\sin\left(\omega p/2\right)}{\sin\left(\omega q/2\right)}\sin\left(q\theta_{0}\right)\right\}/\sigma_{r\theta }^{II}\left(\theta =0\right)\right]=1$$

where $K_{\mathrm{Ic}}^{SV}$ is the notch fracture toughness obtained from the fracture tests on SV-notched samples under pure mode I loading. Besides, the fracture initiation angle *θ*_0_ in Equation (9) is determined from the following formula derived by Ayatollahi et al. [8], derived from differentiation of the tangential stress relative to angle *θ* and combinations with Equations (3) and (4):

(10) $$Y_{I}^{SV}\left[\left\{m\sin\left(m\theta_{0}\right)-m\frac{\sin\left(\omega m/2\right)}{\sin\left(\omega n/2\right)}\sin\left(n\theta_{0}\right)\right\}/\sigma_{\theta \theta }^{l}\left(\theta =0\right)\right]+\left({\left(d\right)}^{\lambda_{1}-\lambda_{2}}Y_{\mathrm{II}}^{SV}\right){\left(r_{c,V}\right)}^{t}\left[\left\{-p\cos\left(p\theta_{0}\right)+q\frac{\sin\left(\omega p/2\right)}{\sin\left(\omega q/2\right)}\cos\left(q\theta_{0}\right)\right\}/\sigma_{r\theta }^{II}\left(\theta =0\right)\right]=0$$

The critical distance *r_c_*_,*V*_ in both Equations (9) and (10) can be determined from the material fracture toughness *K_Ic_* and tensile strength *f_t_*, applying the formulation proposed by Carpinteri et al. as [27]:

(11) $$r_{c,V}=\frac{1}{2\pi }{\left(\frac{K_{Ic}}{f_{t}}\right)}^{2}$$

The previous formulation is applicable to linear-elastic brittle materials. In quasi-brittle materials such as rocks, the critical distance *r_c_*_,*V*_ is large when compared to the specimen size and, therefore, on many occasions, classical fracture criteria like SV-MTS, which takes into account only the singular terms of stress field, cannot predict accurately the fracture load of rock notched samples. In such cases, it has been suggested in previous studies that higher order terms in the stress field could be taken into consideration [28,29,30,31,32,33,34] or, alternatively, the stress or strain components could be determined directly from FEM analyses. The MTS-FEM approach, utilized recently for predicting the fracture behavior of rock notched samples [22,35,36,37], corresponds to the second approach. It is based on the MTS criterion, in which the tangential stress at the critical distance is determined from finite element method. Assuming linear elastic behavior in the finite element analysis, the stress field ahead of the SV-notch tip is proportional to the magnitude of the applied external load. Thus, a simple proportion between the maximum value of tangential stress at the critical distance *r_c_*_,*V*_ obtained from the *FEM*, the corresponding arbitrary load applied in the *FEM*, and the tensile strength *f_t_* provides the fracture load as follows:

(12) $$P_{f}=f_{t}\frac{P_{FEM}}{\sigma_{\theta \theta }\left(r_{c,V},\theta_{0}\right){|}_{FEM}}$$

Here, it should be noted that both *r_c_*_,*V*_ and *θ_0_* are determined from *FEM*. This is mainly because the objective of the approach is the prediction of the fracture resistance of SV-notched samples without computing any stress coefficients.

In this way, the cracked Brazilian disk tested for determining the fracture toughness is simulated by FEM at the corresponding fracture load conditions. Then the distance from the crack tip along the crack line to the point where the tangential stress is equal to the tensile strength is determined, and set as the critical distance *r_c_*. Additionally, the value of critical distance for the cracked and SV-notched samples is assumed to be equal in this study (i.e., it is a material property) as already reported in previous research works (e.g., [37,38]). Indeed, the critical distances for cracked and notched samples may be considered conceptually different because of the influence of both the notch root radius and the notch opening angle (e.g., [39,40,41]. However, there are studies in the literature in which the critical distance is considered the same for both notched and cracked specimens [37,38]. This simplification allows extra experimental work to be avoided and, as justified in the literature (e.g., [37,38]) and below, provides good agreement with the experimental data.

The fracture initiation angle *θ*_0_ is determined by measuring the tangential stress at a ring with radius of *r_c_*_,*V*_ around the notch tip. The direction perpendicular to the maximum tangential stress determines *θ_0_*. In other words, the angle between this direction and the notch bisector line is known as *θ_0_*. Additionally, the value of the tangential stress at *θ*_0_ is substituted into Equation (12) as *σ_θθ_(r_c_*_,*V*_,*θ*_0_*)|_FEM_*. In the next section, details for calculation of *r_c_*_,*V*_ and *σ_θθ_(r_c_*_,*V*_,*θ_0_)|_FEM_* are explained, and the corresponding results are gathered.

## 3. Results

**Figure 7:**
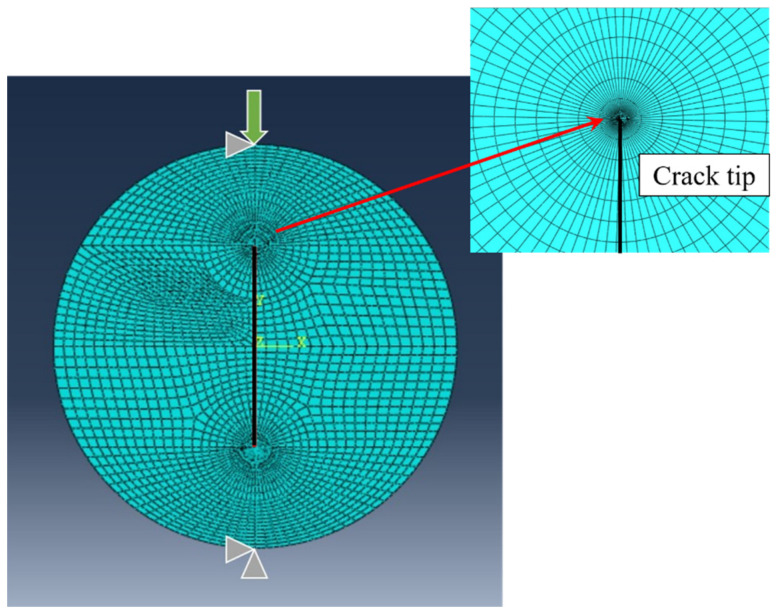
Typical mesh pattern and loading condition utilized for simulating CBD samples.

In both SV-MTS and MTS-FEM criteria, the critical distance *r_c_*_,*V*_ is a key material parameter. When applying the SV-MTS criterion, it is obtained from Equation (11), whereas when applying the MTS-FEM criterion, *r_c_*_,*V*_ is derived from FEM analysis. By substituting the values of *K_Ic_* = 0.977 MPa.√m and *f_t_* = 3.34 MPa into Equation (11), the value of *r_c_*_,*V*_ is calculated as 14 mm. Now, in order to determine the critical distance *r_c_*_,*V*_ in the MTS-FEM approach, the CBD sample was modeled and meshed in Abaqus code with eight-node iso-parametric elements. Then, the singularity at the crack tip was considered by using singular collapsed elements at the first ring around the crack tip. In order to achieve reliable results, three different mesh patterns and sizes were used, and the mesh pattern shown in Figure 7 was finally generated. Furthermore, 10,000 elements were completely generated for CBD samples. The compressive load 17,317 N obtained from fracture tests was applied to the FE model. Figure 7 shows the loading conditions for a CBD sample as well as the generated mesh pattern. After simulation, the tangential stress was measured from the nodes along the crack line. The distance from the crack tip where the tangential stress is equal to 3.34 MPa (the tensile strength of tested rock) is 5 mm, which is considered as *r_c_*_,*V*_.

**Figure 8:**
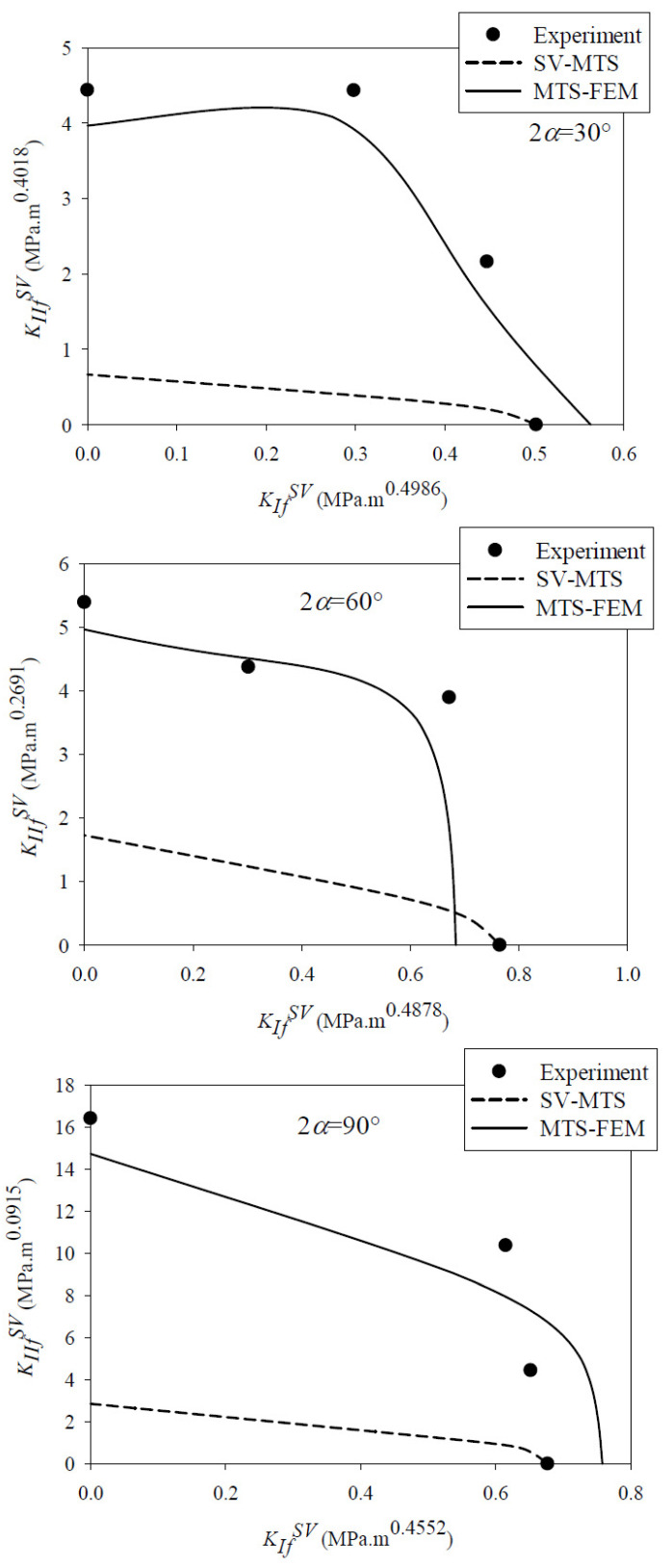
Comparison between the fracture curves of the SVNBD samples predicted by the SV-MTS and the MTS-FEM criteria and the experimental results. Notch opening angles of 2*α* = 30°, 2*α* = 60° and 2*α* = 90°.

When applying the SV-MTS criterion, after determining the value of critical distance *r_c_*_,*V*_, the fracture initiation angle *θ*_0_ is calculated by solving Equation (10). Next, the critical notch stress intensity factors (NSIFs), $K_{\mathrm{If}}^{SV}$ and $K_{\mathrm{IIf}}^{SV}$, are calculated from Equation (9). The results are shown in Figure 8, where they are also compared to the experimental results. A significant discrepancy between the fracture curves obtained from the SV-MTS criterion and the experiments can be observed in the figure, except for the results of pure mode I loading. Since the value of $K_{\mathrm{Ic}}^{SV}$ obtained from fracture tests is used in Equation (9), there are no differences between the theoretical prediction and the experimental result.

**Figure 9:**
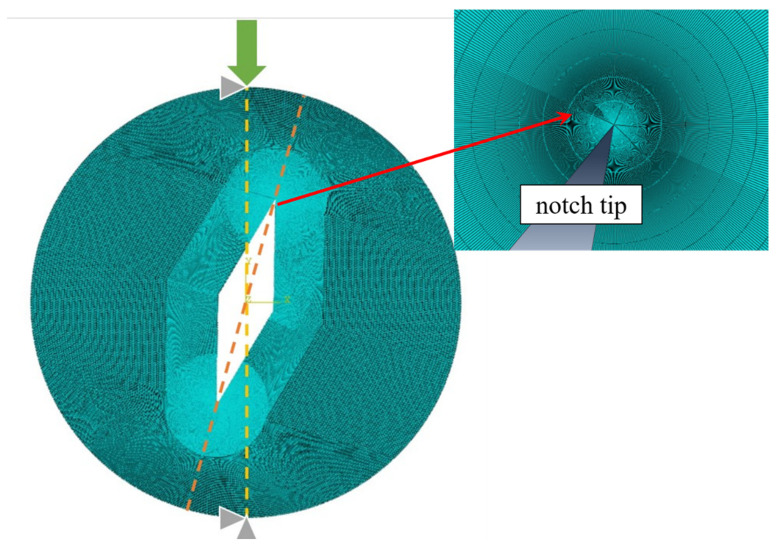
A typical mesh pattern, loading and boundary conditions for the SVNBD samples.

**Figure 10:**
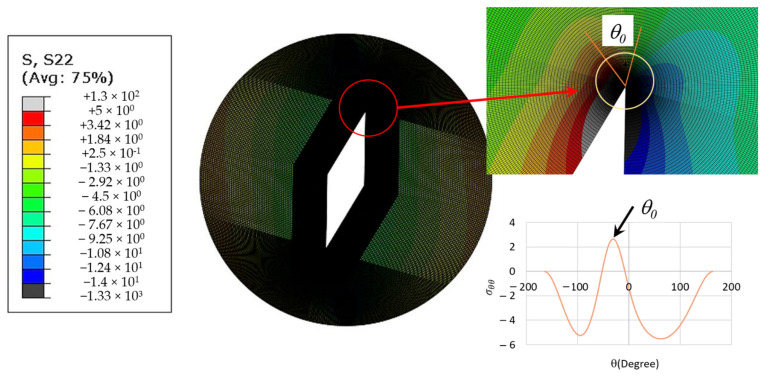
Example of tangential stress results and definition of the location of maximum tangential stress.

As mentioned earlier, calculating the tangential stress from FEM, as proposed in the MTS-FEM approach, can improve the prediction of the conventional MTS criterion for rocks. For this purpose, all SVNBD samples were modeled in Abaqus code and then discretized by nearly 15,000 eight-node iso-parametric elements. Very fine meshes were utilized around the notch tip for considering the high stress gradient in the vicinity of the notch border. It is noteworthy that, similar to the CBD sample, three mesh patterns were utilized first for attaining reliable results, and finally the mesh pattern displayed in Figure 9 was selected. An arbitrary compressive load with magnitude of 10 kN was applied along the diameter of the disk related to the notch bisector line and loading angle *β*. Figure 9 also shows the boundary conditions and the applied load employed in the present modeling of the SVNBD samples, in addition to the mesh pattern. The finite element analysis was performed for each model and then the values of the tangential stress component at a ring with radius of *r_c_*_,*V*_ = 5 mm around the SV-notch tip were measured. The value and orientation of the maximum tangential stress were considered as *σ_θθ_(r_c_*_,*V*_,*θ_0_)|_FEM_* and *θ_0_*, respectively. Figure 10 shows a sample with the resulting tangential stresses around the notch tip and the location of the maximum tangential stress. It is necessary to consider the coordinate transformation rules in second-order tensors due to the difference between the coordinate system used in the finite element models and the conventional coordinate system for SV-notched samples shown in Figure 6. After that, the fracture load for each SVNBD sample was computed by substituting the value of *σ_θθ_(r_c_*_,*V*_,*θ_0_)|_FEM_*, tensile strength of *f_t_* = 3.34 MPa, and applied load of 10,000 N into Equation (12). By replacing the fracture loads predicted by the MTS-FEM criterion in Equations (3) and (4) the fracture curves for each tested rock sample configuration are obtained, with the results being shown in Figure 8. It can be observed that the fracture curves predicted by the MTS-FEM criterion are not only more accurate than those obtained by the SV-MTS criterion, but they are also in agreement with the experimental results.

Moreover, the fracture loads predicted by both the SV-MTS and the MTS-FEM fracture criteria are compared with those obtained in the experimental program, with the results being gathered in Table 3. The value of the fracture load in the SV-MTS criterion is determined by substituting the predicted value of $K_{\mathrm{If}}^{SV}$ or $K_{\mathrm{IIf}}^{SV}$ into Equation (3) or (4), respectively. According to the discrepancy between the theoretical and the experimental fracture loads shown in Table 3, it can be stated again that the MTS-FEM criterion is significantly more accurate than the SV-MTS criterion. In addition, the discrepancy between the fracture loads predicted by the MTS-FEM criterion and the corresponding experiments is nearly 10% for rock SVNBD samples with different notch angles. Therefore, it can be concluded that the MTS-FEM approach can be employed for predicting the onset of fracture in rock-type SV-notched parts. As an additional advantage of the MTS-FEM approach presented in this study, there is no need to calculate the stress coefficients such as NSIFs for predicting the fracture load of rock notched samples.

Moreover, it can be mentioned that the approach derived from the MTS-FEM criterion is independent of the loading angle *β*. In other words, for each loading angle (mixed mode or pure mode II), the tangential stress around the SV-notch tip is determined from FEM, and then, the fracture load is predicted.

## 4. Conclusions

The fracture resistance of rock-type sharp V-notched samples was investigated experimentally and theoretically. Several sharp V-notched Brazilian disks (SVNBD samples) were prepared from white marble sheets and then tested under different mixed mode I/II loading conditions. In order to predict the fracture resistance of the SVNBD samples, the MTS-FEM criterion was utilized. This criterion is based on the maximum tangential stress criterion in which the tangential stress around the SV-notch tip is determined from finite element analysis. Moreover, the critical distance in this approach is obtained from the finite element analysis of the cracked Brazilian disk sample under pure mode I loading. The fracture loads predicted by the present MTS-FEM criterion, as well as those predicted by the previously proposed SV-MTS criterion, were compared to those obtained in the experimental program. The results showed that the MTS-FEM criterion is not only able to predict the fracture resistance of rock-type SV-notched samples more accurately than the conventional SV-MTS criterion, but also that its predictions are in agreement with the experimental results. This improvement is due to the tangential stresses in the MTS-FEM criterion being determined from FEM, providing more accurate stresses than those derived analytically, which are used in the conventional SV-MTS criterion. Furthermore, it is not necessary to calculate the stress coefficients for predicting the fracture loads of rock notched samples, as is the case with the notch stress intensity factors in the MTS-FEM approach.

## Figures and Tables

**Table 1:** Specimen dimensions, loading conditions, and fracture loads for the rock SVNBD samples tested in the present study.

*D* (mm)	*t* (mm)	*d* (mm)	2*α* (°)	*β* (°)	*P_f_* Avg. (N)	Standard Deviation SD (N)
150	29.4	75	30	0	6335.7	646.9
150	32	75	30	8	8081.3	1725.0
150	27.7	75	30	16	8572.4	1692.7
150	30.4	75	30	25	7263.7	1307.7
150	31.4	75	60	0	6599.7	857.7
150	33.3	75	60	9	7096.3	1228.3
150	35.5	75	60	18	5732.4	1307.0
150	34.4	75	60	28	5743.8	2612.6
150	33.9	75	90	0	2903.5	274.7
150	34.5	75	90	11	3421.2	706.3
150	32.8	75	90	22	5283.6	638.7
150	34.6	75	90	34	7396.1	813.4

**Table 2:** Eigenvalues *λ*_1_ and *λ*_2_, dimensionless parameters $Y_{I}^{SV}$ and $Y_{\mathrm{II}}^{SV}$, and the values of parameters $K_{\mathrm{If}}^{SV}$ and $K_{\mathrm{IIf}}^{SV}$ for tested SVNBD samples.

2α (°)	β (°)	$P_{f}$ **Avg.** **(N)**	$\lambda_{1}$	$\lambda_{2}$	$Y_{I}^{SV}$	$Y_{II}^{SV}$	$K_{If}^{SV}$	$K_{IIf}^{SV}$
30	0	6335.7	0.5014	0.5982	1.27	0	0.502 (MPa·m^0.4986^)	0 (MPa·m^0.4018^)
	8	8081.3	0.5014	0.5982	0.97	3.65	0.447 (MPa·m^0.4986^)	2.163 (MPa·m^0.4018^)
	16	8572.4	0.5014	0.5982	0.53	6.13	0.298 (MPa·m^0.4986^)	4.436 (MPa·m^0.4018^)
	25	7263.7	0.5014	0.5982	0	7.85	0 (MPa·m^0.4986^)	4.44 (MPa·m^0.4018^)
60	0	6599.7	0.5122	0.7309	1.94	0	0.765 (MPa·m^0.4878^)	0 (MPa·m^0.2691^)
	9	7096.3	0.5122	0.7309	1.65	5.43	0.672 (MPa·m^0.4878^)	3.895 (MPa·m^0.2691^)
	18	5732.4	0.5122	0.7309	1	8.21	0.302 (MPa·m^0.4878^)	4.374 (MPa·m^0.2691^)
	28	5743.8	0.5122	0.7309	0	9.75	0 (MPa·m^0.4878^)	5.391 (MPa·m^0.2691^)
90	0	2903.5	0.5448	0.9085	3.87	0	0.677 (MPa·m^0.4552^)	0 (MPa·m^0.0915^)
	11	3421.2	0.5448	0.9085	3.26	8.65	0.652 (MPa·m^0.4552^)	4.436 (MPa·m^0.0915^)
	22	5283.6	0.5448	0.9085	1.86	12.21	0.615 (MPa·m^0.4552^)	10.365 (MPa·m^0.0915^)
	34	7396.1	0.5448	0.9085	0	14.56	0 (MPa·m^0.4552^)	16.402 (MPa·m^0.0915^)

**Table 3:** Comparison between the fracture load predictions in rock (marble) SVBD samples obtained from SV-MTS and MTS-FEM criteria and experimental results.

			*P_f_* (N)		Dicrepancy (%)	
2α (°)	β (°)	Experiment	SV-MTS	MTS-FEM	SV-MTS	MTS-FEM
30°	0	6335.7	6335.7	7103.3	0.0	12.1
	8	8081.3	3311.1	7566.9	59.0	6.4
	16	8572.4	1401.7	7847.2	83.6	8.5
	25	7263.7	1117.8	6532.1	84.6	10.1
			Mean discrepancy (%)		56.3	9.25
60	0	6599.7	6599.7	5879.6	0.0	10.9
	9	7096.3	3668.5	6557.8	48.3	7.5
	18	5732.4	2325	5858.8	59.4	2.2
	28	5743.8	1813.1	5271.3	68.4	8.2
			Mean discrepancy (%)		45.2	7.2
90	0	2903.5	2903.5	3243.9	0.0	11.7
	11	3421.2	2023.3	3787.2	40.9	10.7
	22	5283.6	1378	4624.2	73.9	12.5
	34	7396.1	1266.2	6645.7	82.9	10.2
			Mean discrepancy (%)		49.4	11.2

## Data Availability

Details of the results of individual tests can be provided by sending request to J.A. (akbardoost@khu.ac.ir).

## Nomenclature

*d*	Notch length in SVNBD specimens
*D*	Diameter of the circular specimens
*f_t_*	Tensile strength
*K_Ic_*	Fracture toughness
*K_I_^SV^, K_II_^SV^*	Sharp V-notch stress intensity factor under mode I and mode II
*K_If_^SV^, K_IIf_^SV^*	Critical values of *K_I_^SV^* and *K_II_^SV^*, respectively
*K_I_**	Dimensionless parameters for stress terms in cracked conditions
*P*	Applied load
*P_f_*	Fracture load
*r_c,V_*	Critical distance for sharp V-notch
*t*	Specimen thickness
*Y_I_^V^, Y_II_^V^*	Dimensionless parameters for stress terms in notched conditions
*α*	Half of the notch opening angle
*β*	Loading angle for Brazilian disk specimen
*β_II_*	Loading angle corresponding to pure mode II loading
*λ_i_*	Eigenvalues
*σ_rθ_*	In-plane shear stress
*σ_θθ_*	Tangential stress
*σ_θθc_*	Critical value of *σ_θθ_*
*θ_0_*	Notch bifurcation angle
*θ_0I_*	Notch bifurcation angle for pure mode I
*θ_0II_*	Notch bifurcation angle for pure mode II
*ω*	Notch solid angle
*BD*	Brazilian disk
*CBD*	Cracked Brazilian disk specimen
*MTS*	Maximum tangential stress criterion
*MTS-FEM*	Modified MTS criterion based on the finite element method
*SVNBD*	Sharp V-notched Brazilian disk
*SV-MTS*	MTS criterion for sharp V-notch
